# Effectiveness of immediate contrast-enhanced evaluation after endoscopic ultrasound-guided ethanol injection for a pancreatic neuroendocrine tumor

**DOI:** 10.1055/a-2767-1673

**Published:** 2026-01-20

**Authors:** Shuntaro Mukai, Atsushi Sofuni, Takayoshi Tsuchiya, Ryosuke Tonozuka, Kazumasa Nagai, Noriyuki Hirakawa, Takao Itoi

**Affiliations:** 1 Department of Gastroenterology and Hepatology, Tokyo Medical University, Tokyo, Japan

The efficacy of local ablation therapies such as endoscopic ultrasound-guided ethanol injection (EUS-EI) and radiofrequency ablation for small pancreatic neuroendocrine tumors (P-NETs, ≤15 mm, G1) has been reported 1 2 3 4 5 . However, the risk of recurrence due to residual tumor where treatment is inadequate remains a major issue. Here, we report a case showing the effectiveness of immediate contrast-enhanced EUS evaluation after EUS-EI for a small P-NET as a means of overcoming this issue.

**Fig. 1:**
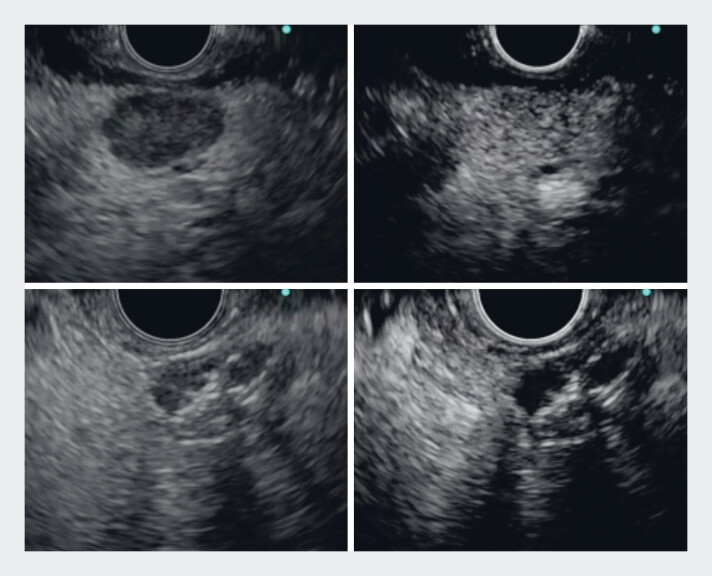
Contrast-enhanced endoscopic ultrasound (CE-EUS) findings before and after endoscopic ultrasound-guided ethanol injection (EUS-EI). (Top) The pancreatic tumor showed early enhancement on pre-treatment CE-EUS evaluation. (Bottom) Immediately after EUS-EI, early enhancement of the pancreatic tumor almost completely disappeared on CE-EUS evaluation.

**Fig. 2:**
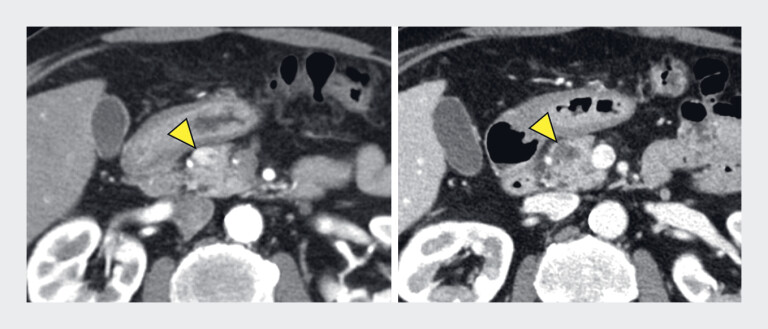
Contrast-enhanced computed tomography (CE-CT) findings before and after EUS-guided ethanol injection (EUS-EI). At the location where the pancreatic tumor was enhanced on CE-CT before EUS-EI (left), no residual tumor was observed after EUS-EI (right), showing that complete ablation was achieved.

A 63-year-old man had a 15-mm pancreatic tumor in the head of the pancreas. The tumor showed early enhancement on contrast-enhanced computed tomography (CE-CT) and contrast-enhanced EUS. A pathological diagnosis of the P-NET (G1) was made by EUS-guided tissue acquisition. The patient declined surgical treatment and requested minimally invasive local ablation therapy using EUS-EI. Three transgastric punctures were performed using a 25-gauge needle, and a total of 1.8 mL of ethanol was injected. Just after EUS-EI, a high-echo area spread within the tumor, revealing the injected region. However, after a short time, it appeared as a low-echo area, making it impossible to identify areas where injection was inadequate and residual tumor was present. When contrast-enhanced EUS was subsequently performed using 0.5 mL perfluorobutane (Sonazoid; Daiichi-Sankyo, Tokyo, Japan), the contrast effect in the tumor, which showed early enhancement before EUS-EI, almost completely disappeared immediately after EUS-EI, confirming that there were no areas where the injection was insufficient ( Fig. 1 , Video 1 ). No residual tumor showing enhancement was observed in a CE-CT scan performed 3 days after EUS-EI, confirming complete ablation of the tumor ( Fig. 2 ). Immediate CE-EUS evaluation after EUS-EI can be considered useful for residual tumor evaluation.

The effectiveness of immediate contrast-enhanced endoscopic ultrasound (EUS) evaluation after EUS ethanol injection (EUS-EI) for a small pancreatic neuroendocrine tumor was shown.Video 1

Endoscopy_UCTN_Code_TTT_1AS_2AI
